# Plasma Transfusion in Septic Shock—A Secondary Analysis of a Retrospective Single-Center Cohort

**DOI:** 10.3390/jcm11154367

**Published:** 2022-07-27

**Authors:** Maximilian Dietrich, Tobias Hölle, Lazar Detelinov Lalev, Martin Loos, Felix Carl Fabian Schmitt, Mascha Onida Fiedler, Thilo Hackert, Daniel Christoph Richter, Markus Alexander Weigand, Dania Fischer

**Affiliations:** 1 Department of Anesthesiology, Heidelberg University Hospital, Im Neuenheimer Feld 420, 69120 Heidelberg, Germany; tobias.hoelle@med.uni-heidelberg.de (T.H.); lazardetelinov.lalev@med.uni-heidelberg.de (L.D.L.); felix.schmitt@med.uni-heidelberg.de (F.C.F.S.); mascha.fiedler@med.uni-heidelberg.de (M.O.F.); daniel.richter@med.uni-heidelberg.de (D.C.R.); markus.weigand@med.uni-heidelberg.de (M.A.W.); dania.fischer@med.uni-heidelberg.de (D.F.); 2 Department of General, Visceral and Transplantation Surgery, Heidelberg University Hospital, Im Neuenheimer Feld 420, 69120 Heidelberg, Germany; martin.loos@med.uni-heidelberg.de (M.L.); thilo.hackert@med.uni-heidelberg.de (T.H.)

**Keywords:** plasma transfusion, fresh frozen plasma, sepsis, shock, resuscitation, coagulopathy, septic shock

## Abstract

In sepsis, both beneficial and detrimental effects of fresh frozen plasma (FFP) transfusion have been reported. The aim of this study was to analyze the indication for and effect of FFP transfusion in patients with septic shock. We performed a secondary analysis of a retrospective single-center cohort of all patients treated for septic shock at the interdisciplinary surgical intensive care unit (ICU) of the Heidelberg University Hospital. Septic shock was defined according to sepsis-3 criteria. To assess the effects of FFP administration in the early phase of septic shock, we compared patients with and without FFP transfusion during the first 48 h of septic shock. Patients who died during the first 48 h of septic shock were excluded from the analysis. Primary endpoints were 30- and 90-day mortality. A total of 261 patients were identified, of which 100 (38.3%) received FFP transfusion within the first 48 h after septic shock onset. The unmatched analysis showed a trend toward higher 30- and 90-d mortality in the FFP group (30 d: +7% *p* = 0.261; 90 d: +11.9% *p* = 0.061). In the propensity-matched analysis, 30- and 90-day mortality were similar between groups. Plasma administration did not influence fluid or vasopressor need, lactate levels, ICU stay, or days on a ventilator. We found no significant harm or associated benefit of FFP use in the early phase of septic shock. Finally, plasma should only be used in patients with a strong indication according to current recommendations, as a conclusive evaluation of the risk-benefit ratio for plasma transfusion in septic shock cannot be made based on the current data.

## 1. Background

Rapid fluid resuscitation with the goal of restoring tissue perfusion is considered one of the cornerstones of early sepsis management. Current practice guidelines recommend crystalloids as first-line fluid [1,2]. However, it is a matter of ongoing debate whether crystalloids are the optimal fluid for sepsis resuscitation [3]. Pharmacologically, balanced crystalloids mimic extracellular electrolyte composition, and the majority of intravenously administered crystalloid fluid rapidly shifts into the interstitium. In septic shock, aggressive crystalloid fluid administration aggravates glycocalyx degradation, promotes tissue edema and potentially worsens coagulopathy [4]. There is an association between the amount of infused volume and adverse outcomes in shock states, which is related to increased shedding of the glycocalyx and breakdown of adherent junctions and tight junctions. The result is increased leakage across the hyperpermeable endothelium [5]. Additional albumin administration as colloid fluid is recommended by current guidelines in patients who require a high amount of crystalloid resuscitation fluid for stabilization. Synthetic colloids as hydroxy-ethyl-starch or gelatin, on the other hand, should be avoided in septic patients [6].

Animal studies and observational data suggest that plasma transfusion not only limits glycocalyx damage but might be able to reverse damage in critically ill patients and patients with septic shock [7,8,9]. Furthermore, plasma exchange has been investigated as a rescue procedure for refractory septic shock, indicating a reduction in mortality and improved recovery from organ failure [10,11].

However, current hemotherapy guidelines only recommend fresh frozen plasma (FFPs) in cases of massive transfusion or for the correction of coagulopathy that cannot be treated with factor concentrates alone [12]. When considering the transfusion of FFP in septic patients beyond these indications and, specifically, for primary volume replacement, potential risks and adverse effects need to be considered. The risk of transmitting infectious diseases through FFP transfusion has been reduced considerably by donor selection, testing, and quarantining, but a measurable risk for transmitting infectious diseases remains [13,14]. Huisman et al. reported a transmission rate of 0.02 per 10,000 FFP transfusions for both human immunodeficiency virus (HIV) and hepatitis C virus (HCV) [15]. Additionally, newly emerging pathogens might develop into further threats (as was the initial fear with SARS-CoV-2 or Zika-Virus). To further minimize the risk of transfusion-transmitted diseases, virus-inactivated plasma products are available, but availability and frequency of application vary between countries [16].

Another potential side effect is transfusion associated circulatory overload (TACO), which is gaining increased relevance and attention, although it is presumably still severely underdiagnosed and underreported, with a stated incidence of approximately 1% in the aftermath of plasma transfusions [17,18,19,20,21]. In a cohort of mixed intensive care unit patients who received transfusions, TACO was diagnosed in 3.6–5.8%, grossly depending on the indication for transfusion, patient preconditions, and monitoring of volume management [22,23,24]. Allergic reactions may also occur with an approximate incidence rate of 1–3% [25].

Advances in the knowledge of the origin of transfusion-related acute lung injury (TRALI) have led to changes in the guidelines for plasma collection, which have considerably reduced the risk of TRALI from plasma transfusion [26,27,28]. Nevertheless, TRALI still has an estimated incidence rate of 0.02 to 1.12% per transfused blood product and can exceed 5 to 8% in critically ill adults and children, with a mortality rate of up to 43% in these high risk patients [29].

Plasma transfusion may furthermore increase the risk of nosocomial infections in parallel with the development of immune modulation referred to as Transfusion-related immunomodulation (TRIM). In emergency and elective general surgery, trauma and critical care, plasma transfusion was associated with postoperative infectious complications [30,31,32,33,34,35,36]. However, as these studies were of retrospective nature, it cannot be ruled out that the situations leading to the FFP transfusion were the cause for these observations rather than the transfusions themselves.

Furthermore, the effect of FFP transfusion on Sepsis induced coagulopathy (SIC) is quite unpredictable, as plasma contains both pro- and anticoagulant factors. SIC remains a major determinant of mortality in septic patients.

Current Surviving Sepsis Campaign guidelines for managing sepsis and septic shock do not provide a statement on the use of FFP [1]. It remains unclear whether the potential benefits of plasma outweigh the risks when transfused in the stabilization phase of septic shock.

In this secondary analysis of a retrospective single-center cohort, we aimed to investigate potential effects on mortality, coagulation, and organ failure, as well as the occurrence of side effects of FFP transfusion in septic shock patients with a predominantly abdominal focus in a surgical ICU setting.

## 2. Methods

### 2.1. Study Design

The present study is a secondary analysis of a retrospectively collected clinical database of a before-and-after study [37]. The local Institutional Ethics Committee approved the study (reference number: S-586/2020; study protocol: Version 2.0).

### 2.2. Patient Cohort

Patients treated for septic shock between March 2015 and July 2020 at the interdisciplinary surgical ICU of the Heidelberg University Hospital, Germany, were included. The following criteria were used to define septic shock: Suspected infection, increase in SOFA score ≥2 points, vasopressor requirement to maintain sufficient hemodynamics (MAP ≥ 65 mmHg) despite sufficient volume substitution, serum lactate > 2 mmol/L (18 mg/dL).

To assess the effects of FFP administration in the early phase of septic shock, we compared patients with and without FFP transfusion during the first 48 h of septic shock. Patients who died during the first 48 h of septic shock were excluded from the analysis to reduce heterogeneity of our cohort. The therapy of septic shock patients was determined by the care-giving physicians according to local protocols based on international guidelines [1,38,39].

### 2.3. Data Collection

All data presented in this analysis were derived retrospectively from clinical documentation systems. Demographics, pre-existing conditions, source of infection, indication for plasma transfusion, and the sepsis-induced coagulopathy (SIC) score were obtained. SIC-Score, a prognostic score proposed for the diagnosis of SIC, includes platelet count, International Normalized Ratio (INR), and sepsis-related organ failure assessment (SOFA) score [40]. Vasopressor dependence and inotropic support were summarized and analyzed using the vasoactive-inotropic score (VIS [41]), to allow a better comparison of different vasoactive agents.

VIS = dopamine dose (μg/kg/min) + dobutamine dose (μg/kg/min) + 100 × epinephrine dose (μg/kg/min) + 100 × norepinephrine dose (μg/kg/min) + 10,000 × vasopressin dose (U/kg/min) + 10 × milrinone dose (μg/kg/min)

Leucocyte count, platelet count, INR, C-reactive protein (CRP), procalcitonin (PCT), albumin, creatinine, and lactate levels were collected from the laboratory documentation system. The SOFA score was rated once daily by the care-giving physicians. Laboratory data, clinical scores, and catecholamine support were obtained at study inclusion, 48 h, 96 h, 168 h, and 14 days after study inclusion.

### 2.4. Study Endpoints

The primary outcome parameters were 30- and 90-day mortality. Secondary outcomes included the ICU- and hospital mortality, the ICU length of stay and the duration of invasive ventilation, administration of fluid, packed red blood cells (PRC), and albumin, catecholamine support (VIS), SIC score, INR, CRP, PCT and SOFA score at enrollment and 48 h, 96 h, 7 and 14 days after study inclusion.

### 2.5. Statistical Analysis

Data was collected with the aid of an electronic database system (Microsoft Excel^®^, Microsoft Deutschland GmbH, Unterschleißheim, Germany). SPSS Statistics (Version 28.0.0) was used for statistical analyses. Descriptive statistics were done for the complete dataset. For continuous variables median and interquartile range (IQR) are reported. The Man-Whitney-U test was used for the comparison of metric data between unpaired samples. Survival analysis was performed using a Kaplan-Meier graph with log-rank test and a fixed observation period of 30 and 90 days. Laboratory data and score courses over time were evaluated with a Friedman test. Categorial variables are reported with absolute and relative frequencies and the chi-squared-Test was used. To adjust for baseline differences between patients with and without plasma transfusion for the primary and secondary endpoint analyses, a propensity-score matching was performed including age, sex, lactate level, SOFA- and VIS. Appropriate statistical graphics were used to visualize the findings.

## 3. Results

### 3.1. Baseline Characteristics

A total of 261 patients with septic shock according to the SEPSIS-3 Definition, who survived the first 48 h after the onset of septic shock were included in the analysis. Of those 261 patients, 100 patients (38.3%) received FFP transfusions within the first 48 h after enrollment. These patients were included in the plasma group (PG). 161 patients did not receive FFP in the first 48 h of septic shock and were assigned to the control group (CG). Demographics at baseline revealed heterogeneous samples with significant differences in age (CG 70a (59;77); PG 63a (53;74) *p* = 0.008) and Body-mass-index (BMI) (CG 27.7 (24.4;32.3); PG 26.5 (23.4;30.2); *p* = 0.043). Parameters of septic shock differed between the groups: SOFA score, VIS, and lactate were significantly higher in the PG indicating a higher disease severity. The PG received a median of 2300 mL (1200; 3850) of FFP. Compromised coagulation (40.4% *n* = 40/99) and bleeding (43.4% *n* = 43/99) were the most common indications. The abdomen was the most frequent focus of infection in both groups (CG 68.9% *n* = 111/161, PG 81% *n* = 81/100). Surgical source control was performed in 77% of the CG (*n* = 124/161) and 88% of the PG (*n* = 88/100). Further information on microbiological data and information about antimicrobial therapy is presented in Supplement Table S1. A propensity score matching was performed for primary and secondary outcome analysis comparing 71 of the respective groups. Lactate was higher and surgical source control was performed more often in the propensity score matched PG. Baseline characteristics for the matched and unmatched samples are shown in Table 1.

### 3.2. Most Common Indication for FFP Transfusion Was Active Bleeding

#### 3.2.1. Unmatched Cohort

**Figure 1:**
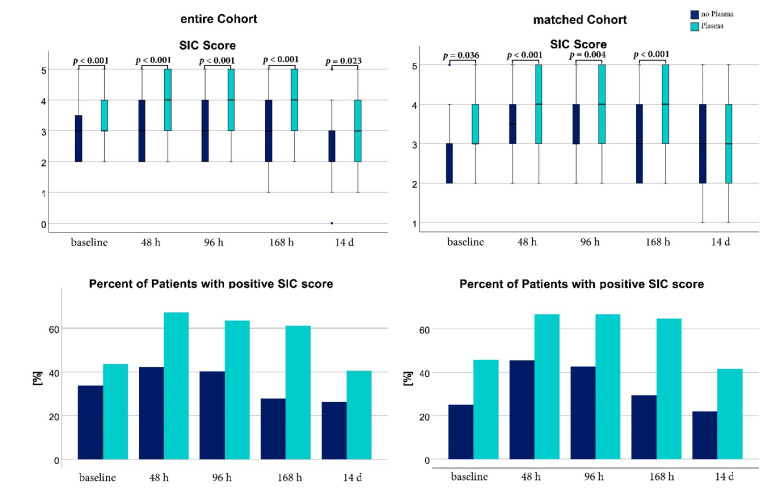
Sepsis-Induced Coagulopathy (SIC) score at different points in time of the matched and the unmatched cohort. Boxplots show the SIC Score for patients who received Plasma (cyan) or no plasma (dark blue) of the matched cohort and unmatched cohort. A Mann-Whitney-U test was used or comparison. The bottom graphics show the relative frequency of patients meeting the cut-off of four or more points in the SIC Score. [%]: percent; [h]: hours; [d]: days.

SIC scores were significantly higher in the PG from baseline up to 168 h after baseline. Absolute SIC scores and the relative number of patients meeting the cut-off of ≥4 points of the SIC score are shown in Figure 1. Platelet count decreased significantly from baseline to 48 h and 96 h after enrollment in both groups. Comparing both groups, platelets were significantly lower in the PG at all measurements. INR was significantly higher in the PG from baseline up to 168h. Main indication for plasma in the PG was bleeding (43.4% *n* = 43/99) or high INR (40.4% *n* = 40/99). In line with this, the PG received a significantly higher median volume of PRCs within the first 48 h after enrollment (CG 0 mL (0; 600); PG 600 mL (0; 1200); *p* < 0.001).

#### 3.2.2. Matched Cohort

**Figure 2:**
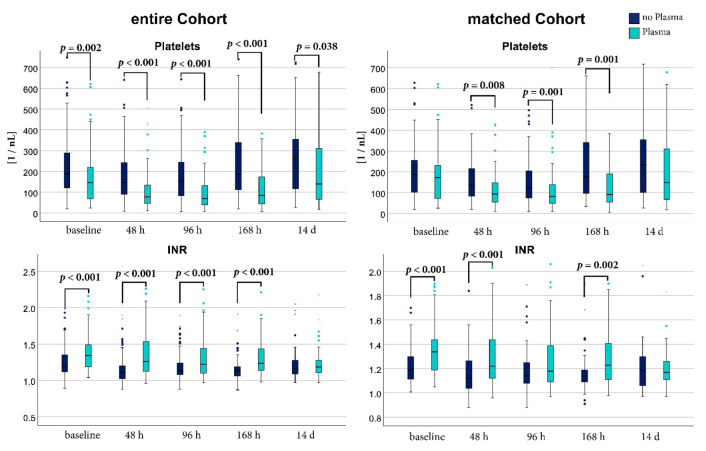
Global parameters of coagulopathy at different points in time of the unmatched and the matched cohort. Platelet count is reported in units per nanoliter [1/nL], international normalized ratio (INR) is reported without unit. A Mann-Whitney-U test was used or comparison between the plasma group (cyan) and the control group (dark blue). [h]: hours; [d]: days.

In the propensity matched analysis, the PG group also had significantly higher SIC scores except at 14 d after inclusion. The platelet decrease over time did not reach significant levels for the CG compared to baseline and for the PG only the comparison from baseline to 168 h reached statistical significance. Platelets at 48 h, 96 h, and 168 h were significantly lower in the PG compared to the CG. INR at baseline, 48 h, and 168 h was significantly higher in the PG than in the CG. Analog to the unmatched group, main indication was bleeding (42.8% *n* = 30/70) and a high INR (40% *n* = 28/70). Until 48 h after enrollment, the matched PG received a higher amount of PRCs in comparison to the CG (CG 0 mL (0; 600); PG 600 mL (0; 1500); *p* = 0.001). INR and platelet count for matched and unmatched groups are shown in Figure 2. Exact values of SIC scores, INR and platelet count for the matched and unmatched groups are presented in Supplement Table S2.

### 3.3. FFP Transfusions in the Early Phase of Septic Shock Had No Effect on Mortality

**Figure 3:**
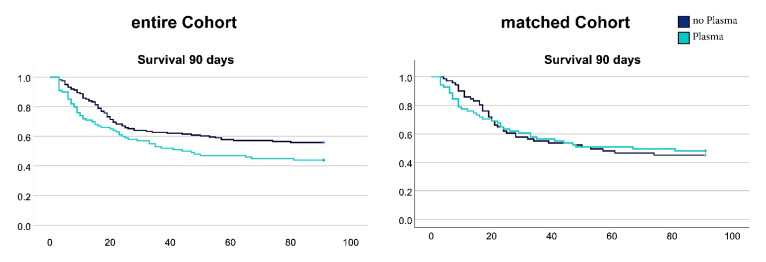
Ninety-day survival plots for the unmatched and the matched cohort. Plots show the relative survival of patients in the plasma group (cyan) and the control group (dark blue) over time. Log-rank test was used for statistical analysis.

The unmatched analysis showed a trend toward higher 30- and 90-d mortality in the PG (30 d: +7% *p* = 0.261; 90 d: +11.9% *p* = 0.061). In the propensity-matched analysis 30- and 90-d mortality were similar between PG and CG. Kaplan-Meier-Plots for the matched and unmatched 90-d survival are shown in Figure 3.

### 3.4. Neither Duration of Invasive Ventilation Nor ICU Stay Were Significantly Influenced by Plasma Administration

#### 3.4.1. Unmatched Cohort

Patients who received plasma had a significantly longer duration of invasive ventilation (CG 6 d (3;14); PG 9,5d (4;18); *p* = 0.006), whereas no significant difference in the duration of ICU stay was observed (CG 10 d (6;19); PG 12 d (6;24); *p* = 0.119).

#### 3.4.2. Matched Cohort

In the matched analysis both ICU stay (CG 14 d (8;21); PG 16 d (7;24); *p* = 0.813) and the duration of invasive ventilation (CG 10 d (4;18); PG 10 d (5;20); *p* = 0.707) did not differ significantly between the CG and the PG.

### 3.5. Plasma Did Not Lead to Faster Hemodynamic Stabilization and Recovery from Organ Dysfunction

#### 3.5.1. Unmatched Cohort

**Figure 4:**
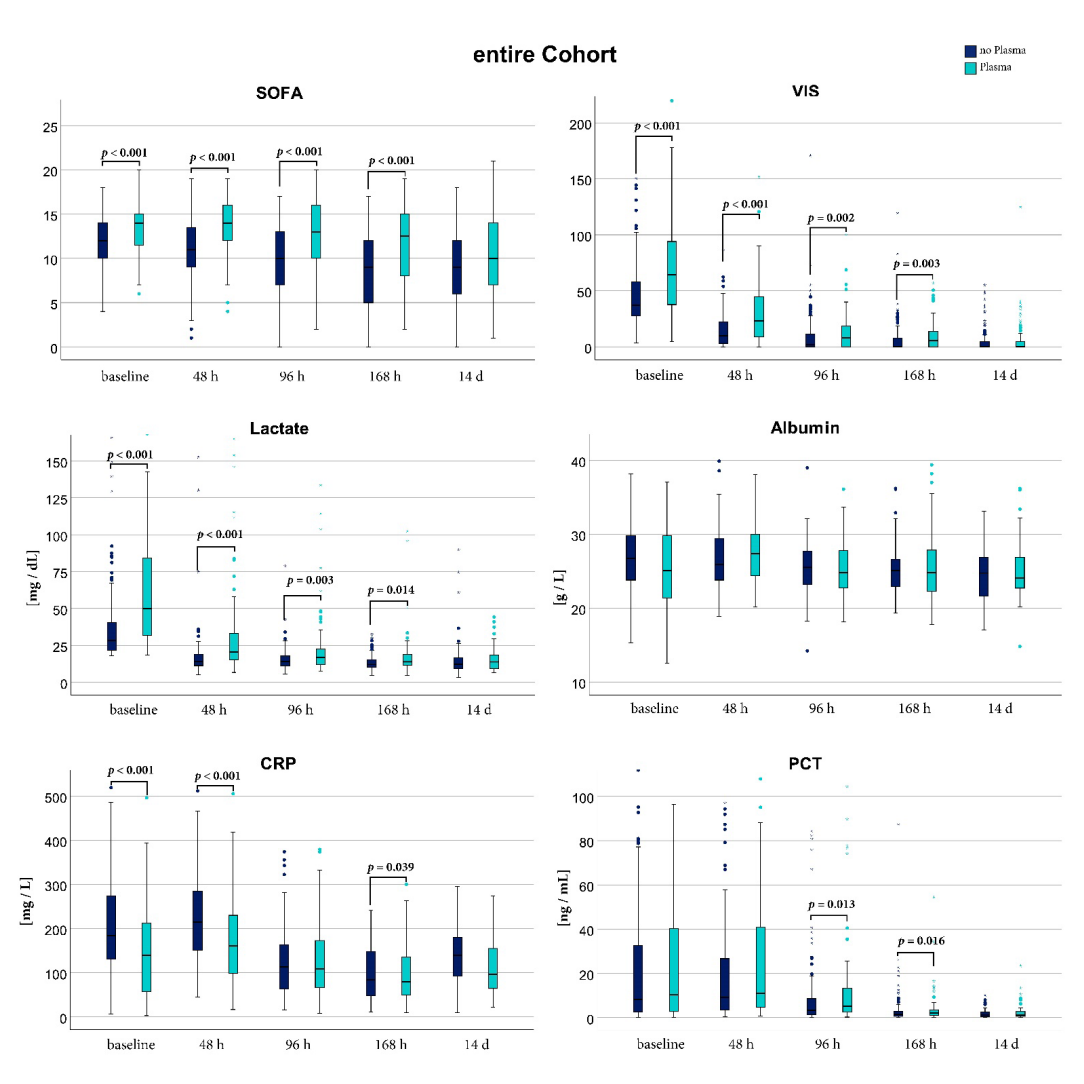
Septic shock related parameters at different points in time of the entire cohort. Comparison of the plasma group (cyan) and the control group (dark blue) was performed with a Mann Whitney U test. SOFA: Sepsis related Organ Failure Assessment Score; VIS: Vasoactive Inotropic Score; CRP: C-related Protein; PCT: Procalcitonin; [mg/dL]: milligram per deciliter; [g/L] gram per liter; [mg/L]: milligram per liter; [ng/mL]: nanogram per milliliter [h]: hours; [d]: days.

Patients of the PG received a higher amount of intravenous fluid per day until 96 h after enrollment, also the administration of intravenous albumin was higher between 48 h and 96 h (Table 2). CRP at the baseline was significantly higher in the CG (CG 184.3 mg/dL (130.8;274.35); PG 139.05 mg/dL (56.95;212.35); *p* = < 0.001), PCT values at baseline showed no significant difference (CG 8.17 ng/mL (2.63; 32.64); PG 10.23 ng/mL (2.73; 40.305); *p* = 0.59). CRP fell over time and aligned after 96 h in both groups. PCT also declined over time, but more gradually, and was significantly higher in the PG at 96 h and 168 h after inclusion. All markers of septic shock and organ failure like SOFA score, VIS, and lactate were significantly higher in the PG compared to the CG up to 168 h after baseline and aligned only after 14 days, suggesting a more pronounced shock in the PG. The courses of SOFA score, VIS, lactate, albumin, CRP, and PCT for the unmatched cohort are shown in Figure 4.

#### 3.5.2. Matched Cohort

**Figure 5:**
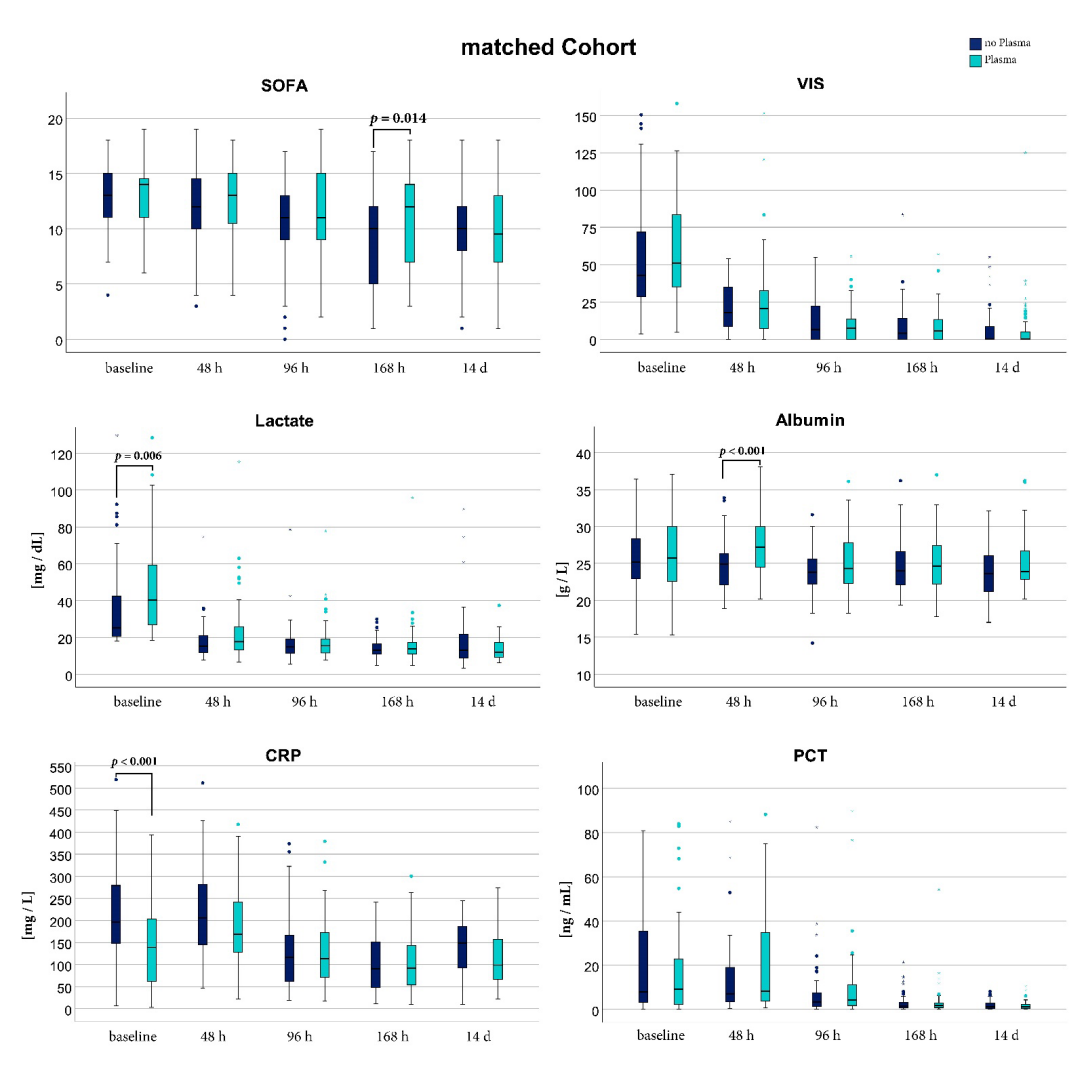
Septic shock related parameters at different points in time of the matched cohort. Comparison of the plasma group (cyan) and the control group (dark blue) was performed with a Mann Whitney U test. SOFA: Sepsis related Organ Failure Assessment Score; VIS: Vasoactive Inotropic Score; CRP: C-related Protein; PCT: Procalcitonin; [mg/dL]: milligram per deciliter; [g/L] gram per liter; [mg/L]: milligram per liter; [ng/mL]: nanogram per milliliter.

In the matched analysis, there were no differences in the given volume of intravenous fluids or albumin (Table 2). CG patients showed a significantly higher CRP at baseline with no difference in PCT. While SOFA score and VIS were comparable in the matched analysis, lactate was significantly higher in the PG at baseline and normalized up until 48 h after enrollment. Patients treated with plasma had significantly higher albumin levels 48 h after enrollment, in the further course no more difference was observable. The courses of SOFA score, VIS, lactate, albumin, CRP, and PCT for the matched cohort are shown in Figure 5.

## 4. Discussion

Studies on the effects of plasma transfusion in patients with a critical illness are limited. Generally, plasma usage is highly variable and therefore affects outcome [42]. To the best of our knowledge, no randomized-controlled trials investigated the use of plasma for fluid resuscitation in septic shock in comparison to crystalloid fluid. In this retrospective analysis, we analyzed the effects of FFP transfusion in the first 48 h of septic shock in a cohort of 261 patients with predominantly abdominal focus. More than one-third of the patients in our cohort received plasma during the first 48 h of septic shock. This could be since we examined a collective with high disease severity treated in an operative intensive care unit. Both surgery and septic shock are associated with an increased risk of bleeding. Accordingly, the main indications for plasma administration were bleeding and coagulation disorders. Furthermore, patients who received plasma suffered from more severe septic shock, as indicated by SOFA score, lactate, fluid and vasopressor dose. This finding corresponds to a study from Denmark, which reported plasma use predominantly in septic patients with more pronounced organ failure [43]. A relevant component of hemorrhagic shock is presumably present in patients with septic shock and bleeding.

Coagulopathy is a predictor of worse outcomes in septic shock and new studies indicate it may promote the transition from sepsis to septic shock [44]. Plasma administration could affect SIC in different ways: the procoagulant factors contained in FFP could potentially aggravate the pathological procoagulant processes, but since FFP contains the full spectrum of pro- and anticoagulant factors, it could potentially affect the equilibrium.

Furthermore, plasma administration and therapeutic plasma exchange has been described to counteract endothelial damage and glycocalyx degradation [9]. In the present study no surrogate parameters of the glycocalyx were available. However, the described positive impact of plasma on the glycocalyx were not reflected by an observable positive influence on clinical outcomes. Patients who received plasma had higher SIC scores at baseline until 168h after enrollment. The SIC score is derived from the SOFA score, platelet count, and INR value. We observed a decrease in platelet count in the plasma group, which could be interpreted as a sign of increased SIC or a consumption by active bleeding or dilution. Plasma transfusion did not have a significant impact on plasmatic coagulation, which is in line with previous studies, that reported no clear benefit of high-dose FFP therapy on any coagulation parameters in patients with massive bleeding [45,46]. The reason for this is that the decisive factor for an effective correction is not the total amount of coagulation factors that are substituted, but their concentration [47]. The concentrations of coagulation factors in FFP correspond at best to physiological levels and are therefore too low to achieve a relevant change in coagulopathic and bleeding patients. Moreover, the fibrinogen level, which is often reduced in bleeding, is diluted rather than increased [45]. Additionally, it has to be kept in mind that both pro- and anticoagulant coagulation factors are present in FFP [48].

For an effective plasma therapy in terms of coagulation, a sufficiently high dose is required that has to be transfused rapidly: a minimum of 15 mL/kg body weight, infusion rate 30–50 mL/min. In adults any dose below 600 mL (2 to 3 units) is inadequate [49]. Additionally, it should be noted that some coagulation factors have a short biological half-life (factor V: 12–15 h; factor VII: 3–6 h). The effect of substitution is not maintained over long periods, so short transfusion intervals are necessary to achieve and maintain hemostatically effective plasma levels. Patients with acquired coagulopathies often show an increased turnover of coagulation factors and inhibitors due to consumption and/or loss or dilution, and consequently, shorten and reduce the effectiveness of the plasma [49]. Hence, FFP transfusion is mostly ineffective in coagulation management but may find use as primary volume replacement. The rapid reversal of shock and restoration of adequate perfusion is a major goal of fluid resuscitation. The clinically established target is a rapid degradation of accumulated lactate back to physiological values. David et al. reported rapid hemodynamic improvement in response to therapeutic plasma exchange in a small randomized-controlled trial in patients with severe septic shock. Therapeutic plasma exchange was performed, exchanging a fixed dose of 12 units of human plasma for FFP [10]. Contrarily, in our present study, the administration of FFP without plasma exchange had no benefit in improving lactate clearance or reducing the need for vasopressors. The lactate and vasopressor support in the unmatched cohort that remained elevated for a longer time can be explained by the initial higher burden of disease, as it is no longer present in the matched cohort. However, the first measurement after baseline was at 48 h after enrollment and at that time values were already close to physiological levels. It is known that excessive amounts of crystalloids in septic shock negatively impact mortality and current guidelines suggest the use of colloids in refractory septic shock. The present analysis did not reveal any evidence of a volume-saving effect due to the administration of plasma.

A recent secondary analysis of a publicly available dataset (MIMIC III) even reported an increased risk of mortality in patients who received FFP in the early phase of sepsis [50]. A different study in patients with septic shock who received plasma observed an elevated incidence of ICU acquired infections, which was accompanied by an increased ICU mortality [51]. The studied patient collective already had an existing life-threatening infection and we found no relevant influence on inflammatory biomarkers. In the present study, higher mortality in patients after plasma administration was observed. This finding could sufficiently be explained by the higher burden of disease at baseline and more pronounced shock, supported by the fact that after matching the patients for disease severity, a difference in mortality was no longer present. The prolonged duration of ventilation and the length of ICU stay can also be explained analogously, as there was no difference between the matched cohorts.

Studies have consistently shown that major transfusion-related adverse side-effects TACO and TRALI are underreported and underrecognized [52,53,54]. This means that, although no harmful side effects were reported, we cannot rule out undetected events of TACO or TRALI.

When transfusing the biologically variable blood product FFP, it may also be relevant that these potentially contain further biologically active substances stemming from the donor. In a pharmacological study, up to 12% of FFPs of a representative sample were contaminated with medications [55]. Whether these contaminations can cause an active effect on the plasma recipient is not known and presumably depends on the dose and clinical setting.

Several limitations have to be considered for the interpretation of the result of this study. First, this is a single-center cohort of surgical ICU patients, which limits the generalizability of the results. In our ICU, it is standard of care to replace albumin in patients who received a high amount of crystalloid resuscitation fluid and in those with hypoalbuminemia. A protective effect on the glycocalyx has also been reported for albumin administration, which may have influenced the results of our analysis [56]. The surgical setting also complicates the distinction between different forms of shock. A confounding influence of bleeding and hemorrhagic shock on the outcome cannot be ruled out. Furthermore, we must consider that we did not differentiate between the indication for plasma administration and transfusion decisions may have been highly dependent on the treating physician. It is possible, that the sicker patients predominantly received FFP and even after adjustment for disease severity, a confounding influence might be present.

## 5. Conclusions

There are several theoretical beneficial or detrimental effects of FFP transfusion in sepsis. In this cohort of septic shock patients with a predominantly abdominal focus, plasma was administered mainly to patients with high disease severity. After propensity-score matching, there was no significant impact on mortality. Plasma administration did not lead to improved shock reversal or reduced fluid and vasopressor demand. Finally, plasma should only be used in patients with a strong indication according to current recommendations, as a conclusive evaluation of the risk-benefit ratio for plasma transfusion in septic shock cannot be made based on the available data. Emerging evidence on potential glycocalyx-stabilizing effects warrants larger prospective, randomized trials comparing different types of fluid for septic shock resuscitation.

## Figures and Tables

**Table 1:** Patient Characteristics: categorical values are given in absolute and relative frequencies, a Chi-Squared-Test was used for analysis.

Baseline Patient Characteristics						
	Unmatched Cohort			Matched Cohort		
	No Plasma	Plasma	*p*-Value	No Plasma	Plasma	*p*-Value
Age	70 (59; 77)	63 (53; 74)	0.008	64 (56; 75)	67 (54; 75)	0.898
Sex (male)	107 (66.5%)	63 (63%)	0.569	45 (63.4%)	44 (62%)	0.604
BMI [kg/m^2^]	27.7 (24.4; 32.3)	26.5 (23.4; 30.2)	0.043	25.9 (22.4; 30.9)	27.1 (23.9; 30.7)	0.476
CAD	40 (24.8%)	25 (25%)		15 (21.1%)	22 (31%)	0.291
Cancer	88 (54.7%)	44 (44%)		38 (53.5%)	32 (45.1%)	0.314
Kidney disease			0.74			0.874
AKI	50 (31.1%)	29 (29%)		16 (22.5%)	20 (28.2%)	
CKD	4 (8.7%)	7 (7%)		4 (5.6%)	4 (5.6%)	
Acute on chronic	24 (14.9%)	12 (12%)		11 (15.5%)	9 (12.7%)	
Organ transplant			0.55			0.543
Kidney	7 (4.3%)	3 (3%)		3 (4.2%)	1 (1.4%)	
Heart	1 (0.6%)	1 (1%)		1 (1.4%)	0 (0%)	
Liver	4 (2.5%)	6 (6%)		4 (5.6%)	5 (7%)	
Bone marrow	1 (0.6%)	0 (0%)				
Focus			0.216			0.092
Pulmonary	27 (16.8%)	7 (7%)		15 (21.1%)	4 (5.6%)	
Abdominal	111 (68.9%)	81 (81%)		49 (69%)	57 (80.3%)	
GUI	12 (7.5%)	6 (6%)		3 (4.2%)	5 (7%)	
Vascular	4 (2.5%)	4 (4%)		1 (1.4%)	3 (4.2%)	
Cardiac	1 (0.6%)	0 (0%)				
Catheter-associated	2 (1.2%)	0 (0%)		1 (1.4%)	0 (0%)	
Unkown	4 (2.5%)	2 (2%)		2 (2.8%)	2 (2.8%)	
Surgical source control	124 (77%)	88 (88%)	0.058	49 (69%)	63 (88.7%)	0.014
Creatinine [mg/dL]	1.73 (1.19; 2.51)	1.575 (1.23; 2.2)	0.198	1.7 (1.19; 2.53)	1.48 (1.1; 1.98)	0.06
Lactate [mg/dL]	28.4 (21.7; 40.5)	50 (31.85; 84.2)	<0.001	25.3 (20.5; 42.8)	40.4 (26.9; 59.4)	0.006
CRP [mg/L]	184 (130; 274)	139.05 (57; 212)	<0.001	196 (147; 281.1)	139 (62; 211)	<0.001
PCT [ng/mL]	8.17 (2.63; 32.64)	10.23 (2.73; 40.305)	0.59	7.85 (3.22; 35.32)	9.15 (2.06; 23.64)	0.572
Apache II	32 (27; 36)	33 (28.5; 36)	0.54	31 (26; 35)	33 (27; 38)	0.11
SOFA	12 (10; 14)	14 (11.5; 15)	<0.001	13 (11; 15)	14 (11; 15)	0.897
VIS	37 (27.6; 58)	64.4 (37.6; 94)	<0.001	42.6 (28.2; 72.8)	51 (34.8; 83.3)	0.198
Leucocytes [1/nL]	14.55 (9.39; 24)	12.87 (5.81; 24.27)	0.105	14.21 (9.52; 23.67)	12.7 (6.88; 26.36)	0.543
Platelets	188 (121; 288)	147 (68; 220)	0.002	185 (99; 256)	172 (71; 231)	0.162
INR	1.19 (1.12; 1.35)	1.35 (1.19; 1.49)	<0.001	1.19 (1.11; 1.31)	1.34 (1.18; 1.45)	<0.001
**Plasma**						
FFP 48 h [mL]		2300 (1200; 3850)		1800 (1200; 3400)		
Indication	for Plasma					
	Bleeding event	43 (43.4%)		30 (42.9%)		
	High INR	40 (40.4%)		28 (40.0%)		
	Fluid resuscitation	16 (16.2%)		12 (17.1%)		

Continuous values are reported as mean and interquartile range, a Mann-Whitney-U test was used or comparison. BMI: Body Mass Index; CAD: Coronary Artery Disease; AKI: Acute Kidney Injury; CKD: Chronic Kidney Disease; GUI: Genitourinary Infection; CRP: C-related Protein; PCT: Procalcitonin; SOFA: Sepsis related Organ Failure Assessment Score; VIS: Vasoactive Inotropic Score; INR: International Normalized Ratio; FFP Fresh Frozen Plasma; [kg/m^2^]: kilogram per square meter; [mg/dL]: milligram per deciliter; [mg/L]: milligram per liter; [ng/mL]: nanogram per milliliter; [1/nL]: count per nanoliter; [mL]: milliliter.

**Table 2:** Fluid balance and amount of fluids for the unmatched and matched cohort. Fluid balance and administered amount of fluids overall are given in milliliter (mL), albumin is reported in gram (g). Values are reported as median and interquartile range, comparison between groups was performed with the Mann Whitney U test. [h]: hours; [d]: day.

entire cohort				
	amount of fluids administered per day (mL)			
	baseline to 48 h	48 h–98 h	98 h–168 h	168 h–14 d
control group	3498 (2035; 4857)	4344 (3645; 5320)	4685 (3953; 5709)	4528 (4006; 5317)
plasma group	4350 (2599; 6139)	5114 (4296; 7189)	4629 (4121; 5710)	5234 (3920; 6204)
*p*-value	0.002	<0.001	0.88	0.078
	daily fluid balance (mL)			
	baseline to 48 h	48 h–98 h	98 h–168 h	168 h–14 d
control group	560 (−684; 1640)	−357(−1230; 741)	−432(−1168; 770)	35 (−695; 508)
plasma group	1417 (166; 2838)	44 (−1193; 2034)	−634(−1455; 270)	1 (−984; 598)
*p*-value	0.001	0.042	0.28	0.83
	amount of albumin administered per day (g)			
	baseline to 48 h	48 h–98 h	98 h–168 h	168 h–14 d
control group	0 (0; 10)	0 (0; 20)	7 (0; 20)	9 (3; 17)
plasma group	0 (0; 20)	10 (0; 30)	13 (0; 27)	11 (3; 20)
*p*-value	0.19	0.017	0.11	0.47
matched cohort				
	amount of fluids administered per day (mL)			
	baseline to 48 h	48 h–98h	98 h–168 h	168 h–14 d
control group	4439 (2910; 5972)	4633 (3973; 5552)	4755 (4141; 5685)	4699 (4094; 5546)
plasma group	4370 (2872; 6820)	4958 (4317; 6438)	4647 (4032; 5374)	4880 (4063; 6075)
*p*-value	0.67	0.13	0.52	0.5
	daily fluid balance (mL)			
	baseline to 48 h	48 h–98 h	98 h–168 h	168 h–14 d
control group	1249 (193; 2755)	−391(−1630; 799)	−902(−1853; −19)	−245(−790; 626)
plasma group	1457 (177; 2772)	−203(−1133; 992)	−871(−1585; 49)	149 (−645; 598)
*p*-value	0.55	0.27	0.61	0.53
	amount of albumin administered per day (g)			
	baseline to 48 h	48 h till 98 h	98 h–168 h	168 h–14 d
control group	0 (0; 20)	0 (0; 10)	7 (0; 27)	9 (3; 17)
plasma group	0 (0; 10)	10 (0; 20)	13 (0; 23)	6 (3; 20)
*p*-value	0.71	0.071	0.81	0.7

## Data Availability

The datasets used and/or analyzed during the current study are available from the corresponding author on reasonable request.

## Supplementary Materials

The following are available online at https://www.mdpi.com/article/10.3390/jcm11154367/s1, Figure S1: Septic shock parameters change compared to baseline in the entire cohort. No statistical analysis was performed, these figures are for reference only. SOFA: Sepsis related Organ Failure Assessment Score; VIS: Vasoactive Inotropic Score, Figure S2: Septic shock parameters change compared to baseline in the matched cohort. No statistical analysis was performed, these figures are for reference only. SOFA: Sepsis related Organ Failure Assessment Score; VIS: Vasoactive Inotropic Score, Table S1: Septic shock parameters change compared to baseline in the matched cohort. No statistical analysis was performed, these figures are for reference only. SOFA: Sepsis related Organ Failure Assessment Score; VIS: Vasoactive Inotropic Score, Table S2: Additional sepsis source and microbiologic information; GUI: Genitourinary infection.

## Abbreviations

Control group (CG), fresh frozen plasma (FFPs), intensive care unit (ICU), transfusion associated circulatory overload (TACO), transfusion-related acute lung injury (TRALI), Plasma group (PG)**,** packed red blood cells (PRCs).

## References

[1] Evans L., Rhodes A., Alhazzani W., Antonelli M., Coopersmith C.M., French C., Machado F.R., Mcintyre L., Ostermann M., Prescott H.C. (2021). Surviving sepsis campaign: International guidelines for management of sepsis and septic shock 2021. Intensive Care Med..

[2] Egi M., Ogura H., Yatabe T., Atagi K., Inoue S., Iba T., Kakihana Y., Kawasaki T., Kushimoto S., Kuroda Y. (2021). The Japanese Clinical Practice Guidelines for Management of Sepsis and Septic Shock 2020 (J-SSCG 2020). J. Intensive Care.

[3] Lat I., Coopersmith C.M., De Backer D., Research Committee of the Surviving Sepsis Campaign (2021). The Surviving Sepsis Campaign: Fluid Resuscitation and Vasopressor Therapy Research Priorities in Adult Patients. Crit. Care Med..

[4] Milford E.M., Reade M.C. (2019). Resuscitation Fluid Choices to Preserve the Endothelial Glycocalyx. Crit. Care.

[5] Jaffee W., Hodgins S., McGee W.T. (2018). Tissue Edema, Fluid Balance, and Patient Outcomes in Severe Sepsis: An Organ Systems Review. J. Intensive Care Med..

[6] Perner A., Haase N., Guttormsen A.B., Tenhunen J., Klemenzson G., Aneman A., Madsen K.R., Møller M.H., Elkjær J.M., Poulsen L.M. (2012). Hydroxyethyl starch 130/0.42 versus Ringer’s acetate in severe sepsis. N. Engl. J. Med..

[7] Kozar R.A., Peng Z., Zhang R., Holcomb J.B., Pati S., Park P., Ko T.C., Paredes A. (2011). Plasma restoration of endothelial glycocalyx in a rodent model of hemorrhagic shock. Anesth. Analg..

[8] Chang R., Holcomb J.B., Johansson P.I., Pati S., Schreiber M.A., Wade C.E. (2018). Plasma Resuscitation Improved Survival in a Cecal Ligation and Puncture Rat Model of Sepsis. Shock.

[9] Straat M., Muller M.C., Meijers J.C., Arbous M.S., Spoelstra-de Man A.M., Beurskens C.J., Vroom M.B., Juffermans N.P. (2015). Effect of transfusion of fresh frozen plasma on parameters of endothelial condition and inflammatory status in non-bleeding critically ill patients: A prospective substudy of a randomized trial. Crit. Care.

[10] David S., Bode C., Putensen C., Welte T., Stahl K., The EXCHANGE study group (2021). Adjuvant therapeutic plasma exchange in septic shock. Intensive Care Med..

[11] Keith P.D., Wells A.H., Hodges J., Fast S.H., Adams A., Scott L.K. (2020). The therapeutic efficacy of adjunct therapeutic plasma exchange for septic shock with multiple organ failure: A single-center experience. Crit. Care.

[12] Adam E.H., Fischer D. (2020). Plasma Transfusion Practice in Adult Surgical Patients: Systematic Review of the Literature. Transfus. Med. Hemother..

[13] Zou S., Dorsey K.A., Notari E.P., Foster G.A., Krysztof D.E., Musavi F., Dodd R.Y., Stramer S.L. (2010). Prevalence, incidence, and residual risk of human immunodeficiency virus and hepatitis C virus infections among United States blood donors since the introduction of nucleic acid testing. Transfusion.

[14] MacLennan S., Williamson L.M. (2006). Risks of fresh frozen plasma and platelets. J. Trauma.

[15] Huisman E.L., de Silva S.U., de Peuter M.A. (2014). Economic evaluation of pooled solvent/detergent treated plasma versus single donor fresh-frozen plasma in patients receiving plasma transfusions in the United States. Transfus. Apher. Sci..

[16] Rock G. (2011). A comparison of methods of pathogen inactivation of FFP. Vox Sang..

[17] Raval J.S., Mazepa M.A., Russell S.L., Immel C.C., Whinna H.C., Park Y.A. (2015). Passive reporting greatly underestimates the rate of transfusion-associated circulatory overload after platelet transfusion. Vox Sang..

[18] Li G., Rachmale S., Kojicic M., Shahjehan K., Malinchoc M., Kor D.J., Gajic O. (2011). Incidence and transfusion risk factors for transfusion-associated circulatory overload among medical intensive care unit patients. Transfusion.

[19] Rana R., Fernandez-Perez E.R., Khan S.A., Rana S., Winters J.L., Lesnick T.G., Moore S.B., Gajic O. (2006). Transfusion-related acute lung injury and pulmonary edema in critically ill patients: A retrospective study. Transfusion.

[20] Narick C., Triulzi D.J., Yazer M.H. (2012). Transfusion-associated circulatory overload after plasma transfusion. Transfusion.

[21] Thalji L., Thum D., Weister T.J., Weber W.V., Stubbs J.R., Kor D.J., Nemergut M.E. (2018). Incidence and Epidemiology of Perioperative Transfusion-Related Pulmonary Complications in Pediatric Noncardiac Surgical Patients: A Single-Center, 5-Year Experience. Anesth. Analg..

[22] Bosboom J.J., Klanderman R.B., Zijp M., Hollmann M.W., Veelo D.P., Binnekade J.M., Geerts B.F., Vlaar A.P. (2018). Incidence, risk factors, and outcome of transfusion-associated circulatory overload in a mixed intensive care unit population: A nested case-control study. Transfusion.

[23] Dotsch T.M., Dirkmann D., Bezinover D., Hartmann M., Treckmann J.W., Paul A., Saner F.H. (2017). Assessment of standard laboratory tests and rotational thromboelastometry for the prediction of postoperative bleeding in liver transplantation. Br. J. Anaesth..

[24] O’Leary J.G., Greenberg C.S., Patton H.M., Caldwell S.H. (2019). AGA Clinical Practice Update: Coagulation in Cirrhosis. Gastroenterology.

[25] Gilstad C.W. (2003). Anaphylactic transfusion reactions. Curr. Opin. Hematol..

[26] Muller M.C., van Stein D., Binnekade J.M., van Rhenen D.J., Vlaar A.P. (2015). Low-risk transfusion-related acute lung injury donor strategies and the impact on the onset of transfusion-related acute lung injury: A meta-analysis. Transfusion.

[27] Funk M.B., Guenay S., Lohmann A., Henseler O., Heiden M., Hanschmann K.M., Keller-Stanislawski B. (2012). Benefit of transfusion-related acute lung injury risk-minimization measures—German haemovigilance data (2006–2010). Vox Sang..

[28] Lin Y., Saw C.L., Hannach B., Goldman M. (2012). Transfusion-related acute lung injury prevention measures and their impact at Canadian Blood Services. Transfusion.

[29] McVey M.J., Kapur R., Cserti-Gazdewich C., Semple J.W., Karkouti K., Kuebler W.M. (2019). Transfusion-related Acute Lung Injury in the Perioperative Patient. Anesthesiology.

[30] Ming Y., Liu J., Zhang F., Chen C., Zhou L., Du L., Yan M. (2020). Transfusion of Red Blood Cells, Fresh Frozen Plasma, or Platelets Is Associated with Mortality and Infection After Cardiac Surgery in a Dose-Dependent Manner. Anesth. Analg..

[31] Subramanian A., Berbari E.F., Brown M.J., Allen M.S., Alsara A., Kor D.J. (2012). Plasma transfusion is associated with postoperative infectious complications following esophageal resection surgery: A retrospective cohort study. J. Cardiothorac. Vasc. Anesth..

[32] Inaba K., Branco B.C., Rhee P., Holcomb J.B., Blackbourne L.H., Shulman I., Nelson J., Demetriades D. (2010). Impact of ABO-identical vs ABO-compatible nonidentical plasma transfusion in trauma patients. Arch. Surg..

[33] Inaba K., Branco B.C., Rhee P., Blackbourne L.H., Holcomb J.B., Teixeira P.G., Shulman I., Nelson J., Demetriades D. (2010). Impact of plasma transfusion in trauma patients who do not require massive transfusion. J. Am. Coll. Surg..

[34] Sarani B., Dunkman W.J., Dean L., Sonnad S., Rohrbach J.I., Gracias V.H. (2008). Transfusion of fresh frozen plasma in critically ill surgical patients is associated with an increased risk of infection. Crit. Care Med..

[35] Shah S., Coppolino K., Menocha S., Beceiro S., Nateri J., Spinella P.C., Nicol K., Hall M.W., Muszynski J.A. (2018). Immunomodulatory effects of plasma products on monocyte function in vitro. J. Trauma Acute Care Surg..

[36] Patlan M., Sanchez-Munoz F., Amezcua-Guerra L.M., Granados A., Paez A., Masso F., Mejía A.M., Soster A., Bojalil R., Pavón L. (2017). Effect of fresh frozen plasma on the in vitro activation of U937 monocytes: A potential role for the age of blood donors and their underlying cytokine profile. Biol. Res..

[37] Richter D.C., Dietrich M., Lalev L.D., Schmitt F.C.F., Fiedler M.O., Bruckner T., Stoerzinger D., Chiriac U., Klein S., Hackert T. (2021). Prolonged Infusion of beta-Lactams Decreases Mortality in Patients with Septic Shock: A Retrospective before-and-after Study. Antibiotics.

[38] Rhodes A., Evans L.E., Alhazzani W., Levy M.M., Antonelli M., Ferrer R., Kumar A., Sevransky J.E., Sprung C.L., Nunnally M.E. (2017). Surviving Sepsis Campaign: International Guidelines for Management of Sepsis and Septic Shock: 2016. Crit. Care Med..

[39] Brunkhorst F.M., Weigand M.A., Pletz M., Gastmeier P., Lemmen S.W., Meier-Hellmann A., Ragaller M., Weyland A., Marx G., Bucher M. (2020). [S3 Guideline Sepsis-prevention, diagnosis, therapy, and aftercare: Long version]. Med. Klin. Intensivmed. Notf..

[40] Iba T., Nisio M.D., Levy J.H., Kitamura N., Thachil J. (2017). New criteria for sepsis-induced coagulopathy (SIC) following the revised sepsis definition: A retrospective analysis of a nationwide survey. BMJ Open.

[41] Belletti A., Lerose C.C., Zangrillo A., Landoni G. (2021). Vasoactive-Inotropic Score: Evolution, Clinical Utility, and Pitfalls. J. Cardiothorac. Vasc. Anesth..

[42] Auvinen M.K., Zhao J., Lassen E., Lubenow N., Seger Mollen A., Watz E., Wikman A. (2020). Edgren Patterns of blood use in Sweden from 2008 to 2017: A nationwide cohort study. Transfusion.

[43] Reiter N., Wesche N., Perner A. (2013). The majority of patients in septic shock are transfused with fresh-frozen plasma. Dan. Med. J..

[44] Ren C., Li Y.X., Xia D.M., Zhao P.Y., Zhu S.Y., Zheng L.Y., Liang L.-P., Yao R.-Q., Du X.-H. (2022). Sepsis-Associated Coagulopathy Predicts Hospital Mortality in Critically Ill Patients with Postoperative Sepsis. Front. Med..

[45] Khan S., Davenport R., Raza I., Glasgow S., De’Ath H.D., Johansson P.I., Curry N., Stanworth S., Gaarder C., Brohi K. (2015). Damage control resuscitation using blood component therapy in standard doses has a limited effect on coagulopathy during trauma hemorrhage. Intensive Care Med..

[46] Kujovich J.L. (2005). Hemostatic defects in end stage liver disease. Crit. Care Clin..

[47] Holland L.L., Foster T.M., Marlar R.A., Brooks J.P. (2005). Fresh frozen plasma is ineffective for correcting minimally elevated international normalized ratios. Transfusion.

[48] Yang L., Stanworth S., Hopewell S., Doree C., Murphy M. (2012). Is fresh-frozen plasma clinically effective? An update of a systematic review of randomized controlled trials. Transfusion.

[49] Executive Committee of the German Medical Association on the Recommendation of the Scientific Advisory Board (2016). Cross-Sectional Guidelines for Therapy with Blood Components and Plasma Derivatives: Chapter 5 Human Albumin—Revised. Transfus. Med. Hemotherapy.

[50] Qin X., Zhang W., Zhu X., Hu X., Zhou W. (2021). Early Fresh Frozen Plasma Transfusion: Is It Associated with Improved Outcomes of Patients with Sepsis?. Front. Med..

[51] Peju E., Llitjos J.F., Charpentier J., Francois A., Marin N., Cariou A., Chiche J.-D., Mira J.-P., Lambert J., Jamme M. (2021). Impact of Blood Product Transfusions on the Risk of ICU-Acquired Infections in Septic Shock. Crit. Care Med..

[52] Kopko P.M., Marshall C.S., MacKenzie M.R., Holland P.V., Popovsky M.A. (2002). Transfusion-related acute lung injury: Report of a clinical look-back investigation. JAMA.

[53] Roubinian N. (2018). TACO and TRALI: Biology, risk factors, and prevention strategies. Hematol. Am. Soc. Hematol. Educ. Program.

[54] Skeate R.C., Eastlund T. (2007). Distinguishing between transfusion related acute lung injury and transfusion associated circulatory overload. Curr. Opin. Hematol..

[55] Thevis M., Krug O., Geyer H., Wenzel F., Bux J., Stahl L., Hollmann W., Thom A., Schänzer W. (2013). Monitoring drug residues in donor blood/plasma samples using LC-(MS)/MS—A pilot study. Drug Test. Anal..

[56] Uchimido R., Schmidt E.P., Shapiro N.I. (2019). The glycocalyx: A novel diagnostic and therapeutic target in sepsis. Crit. Care.

