# Supplementary material for: Plasma Transfusion in Septic Shock—A Secondary Analysis of a Retrospective Single-Center Cohort
Source: J Clin Med. 2022 Jul 27;11(15):4367. doi: 10.3390/jcm11154367 (PMC9369152; doi:10.3390/jcm11154367)
Supplement: Supplementary file 1 [file jcm-11-04367-s001.zip › jcm-1786251-supplementary.pdf]

Supplement Table S1. Sepsis-Induced Coagulopathy (SIC) score ,platelets and international normalized ratio (INR) numeric values.

entire cohort

	SIC baseline	SIC 48h	SIC 96h	SIC 168h	SIC 14d
no Plasma	3 (2 ;4)	3 (2 ;4)	3 (2 ;4)	2 (2 ;4)	3 (2 ;3)
Plasma	3 (3 ;5)	4 (3 ;5)	4 (3 ;5)	4 (3 ;5)	3 (2 ;4)

	INR baseline	INR 48h	INR 96h	INR 168h	INR 14d
no Plasma	1,19 (1,12 ;1,35)	1,11 (1,025 ;1,2)	1,135 (1,08 ;1,24)	1,135 (1,06 ;1,19)	1,16 (1,09 ;1,275)
Plasma	1,345 (1,19 ;1,49)	1,26 (1,12 ;1,54)	1,22 (1,1 ;1,44)	1,235 (1,14 ;1,435)	1,185 (1,105 ;1,275)

	Platelets baseline	Platelets 48h	Platelets 96h	Platelets 168h	Platelets 14d
no Plasma	188 (121 ;288)	146,5 (89,5 ;243)	152 (83 ;244)	186,5 (113 ;340)	263 (116,5 ;354,5)
Plasma	146,5 (68 ;219,5)	77 (44 ;134)	70 (40 ;132)	84 (44,5 ;173,5)	140 (65 ;310,5)

matched cohort

	SIC baseline	SIC 48h	SIC 96h	SIC 168h	SIC 14d
no Plasma	3 (3 ;4)	3 (2 ;4)	3 (2 ;4)	2 (2 ;4)	3 (2 ;4)
Plasma	3 (3 ;4)	4 (3 ;5)	4 (3 ;5)	4 (3 ;5)	3 (2 ;4)

	INR baseline	INR 48h	INR 96h	INR 168h	INR 14d
no Plasma	1,19 (1,11 ;1,31)	1,12 (1,04 ;1,27)	1,14 (1,08 ;1,25)	1,14 (1,09 ;1,19)	1,185 (1,06 ;1,3)
Plasma	1,34 (1,18 ;1,45)	1,22 (1,12 ;1,44)	1,18 (1,09 ;1,4)	1,23 (1,11 ;1,41)	1,17 (1,11 ;1,26)

	Platelets baseline	Platelets 48h	Platelets 96h	Platelets 168h	Platelets 14d
no Plasma	185 (99 ;256)	135 (82 ;216)	123 (75 ;204)	176 (97 ;340)	233,5 (101 ;354)
Plasma	172 (71 ;231)	92,5 (55 ;148)	83 (48 ;139)	90,5 (54 ;190)	149 (67 ;310)

Supplement Table S2. Additional sepsis source and microbiologic information; GUI: Genitourinary infection.

		unmatched cohort		matched cohort	
Focus		no Plasma	Plasma	no Plasma	Plasma
	pulmonary	27 (16,8%)	7 (7%)	15 (21,1%)	4 (5,6%)
	abdominal	111 (68,9%)	81 (81%)	49 (69%)	57 (80,3%)
	GUI	12 (7,5%)	6 (6%)	3 (4,2%)	5 (7%)
	vascular	4 (2,5%)	4 (4%)	1 (1,4%)	3 (4,2%)
	unkown	4 (2,5%)	2 (2%)	2 (2,8%)	2 (2,8%)
	catheter associated	2 (1,2%)	0 (0%)	1 (1,4%)	0 (0%)
	cardiac	1 (0,6%)	0 (0%)	17 (23,9%)	5 (7%)
		no Plasma	Plasma	no FFP	FFP
positive source control		134 (83,2%)	91 (91%)	54 (76,1%)	66 (93%)
surgical source control		124 (77%)	88 (88%)	49 (69%)	63 (88,7%)
previous antibiotic treatment		71 (44,1%)	37 (37%)	23 (32,4%)	14 (19,7%)
primary positive sample		no Plasma	Plasma	no Plasma	Plasma
	no positive sample	10 (6,2%)	8 (8%)	4 (5,6%)	3 (4,2%)
	blood culture	62 (38,5%)	26 (26%)	27 (38%)	21 (29,6%)
	tracheal sample	36 (22,4%)	13 (13%)	17 (23,9%)	11 (15,5%)
	wound swab	4 (2,5%)	7 (7%)	4 (5,6%)	5 (7%)
	drain sample	24 (14,9%)	14 (14%)	8 (11,3%)	14 (19,7%)
	rectal/ fecal sample	3 (1,9%)	7 (7%)	2 (2,8%)	2 (2,8%)
	intraoperative swab	17 (10,6%)	21 (21%)	8 (11,3%)	12 (16,9%)
	catherter tip	3 (1,9%)	2 (2%)	0 (0%)	1 (1,4%)
	urin	2 (1,2%)	2 (2%)	1 (1,4%)	2 (2,8%)
primary identified germ		no Plasma	Plasma	no Plasma	Plasma
	eschericha coli	49 (30,4%)	22 (22%)	23 (32,4%)	14 (19,7%)
	klebsiella peumoniae	14 (8,7%)	5 (5%)	6 (8,5%)	5 (7%)
	staphylococcus aures	10 (6,2%)	4 (4%)	3 (4,2%)	4 (5,6%)
	enterococcus faecium	15 (9,3%)	9 (9%)	9 (12,7%)	6 (8,5%)
	VRE	3 (1,9%)	12 (12%)	2 (2,8%)	9 (12,7%)
	peusomonas aeruginosa	8 (5%)	4 (4%)	3 (4,2%)	2 (2,8%)
	other	52 (32,3%)	36 (36%)	21 (29,6%)	28 (39,4%)
secondary positive sample		no Plasma	Plasma	no Plasma	Plasma
	blood culture	24 (14,9%)	8 (8%)	9 (12,7%)	8 (11,3%)
	tracheal sample	33 (20,5%)	11 (11%)	16 (22,5%)	8 (11,3%)
	wound swab	10 (6,2%)	6 (6%)	6 (8,5%)	3 (4,2%)
	drain sample	22 (13,7%)	8 (8%)	9 (12,7%)	8 (11,3%)
	rectal/ fecal sample	6 (3,7%)	4 (4%)	0 (0%)	2 (2,8%)
	intraoperative swab	23 (14,3%)	17 (17%)	11 (15,5%)	11 (15,5%)
	catherter tip	3 (1,9%)	2 (2%)	1 (1,4%)	1 (1,4%)
	urin	4 (2,5%)	6 (6%)	1 (1,4%)	4 (5,6%)
secondary identified germ		no Plasma	Plasma	no Plasma	Plasma
	eschericha coli	18 (11,2%)	12 (12%)	10 (14,1%)	8 (11,3%)
	klebsiella peumoniae	8 (5%)	2 (2%)	2 (2,8%)	1 (1,4%)
	staphylococcus aures	3 (1,9%)	2 (2%)	0 (0%)	2 (2,8%)
	enterococcus faecium	15 (9,3%)	6 (6%)	8 (11,3%)	5 (7%)
	VRE	14 (8,7%)	4 (4%)	1 (1,4%)	2 (2,8%)
	peusomonas aeruginosa	11 (6,8%)	2 (2%)	9 (12,7%)	2 (2,8%)
	other	57 (35,4%)	34 (34%)	24 (33,8%)	25 (35,2%)
Primary Antibiotics		no Plasma	Plasma	no Plasma	Plasma
	Piperacillin	57 (35,4%)	19 (19%)	24 (33,8%)	17 (23,9%)
	Meropenem	93 (57,8%)	68 (68%)	38 (53,5%)	42 (59,2%)
	other	11 (6,8%)	13 (13%)	9 (12,7%)	12 (16,9%)

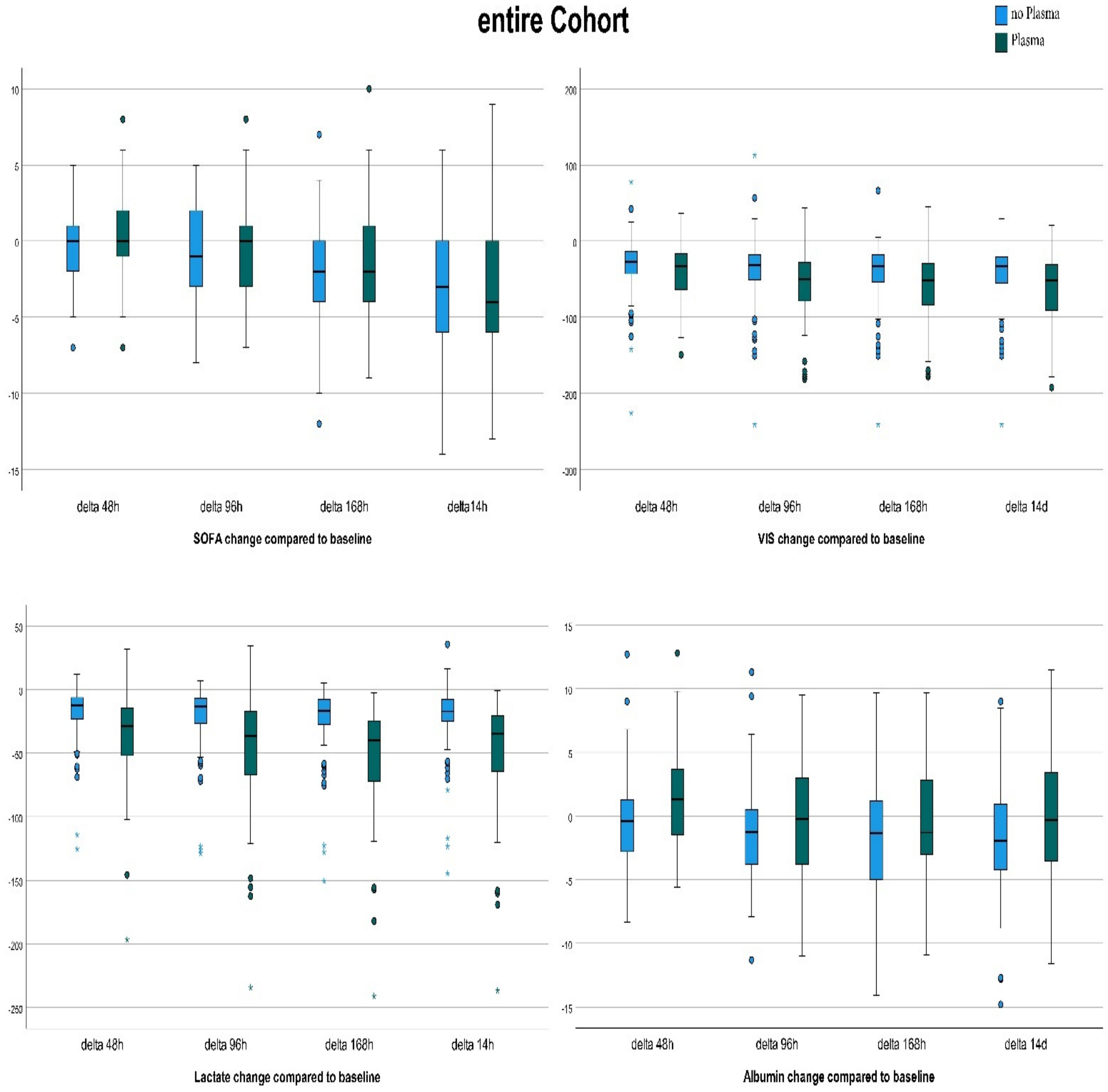

Supplement Figure S1. ***Septic shock parameters change compared to baseline in the entire cohort.*** *No statistical analysis was performed, these figures are for reference only SOFA: Sepsis related Organ Failure Assessment Score; VIS: Vasoactive Inotropic Score.*

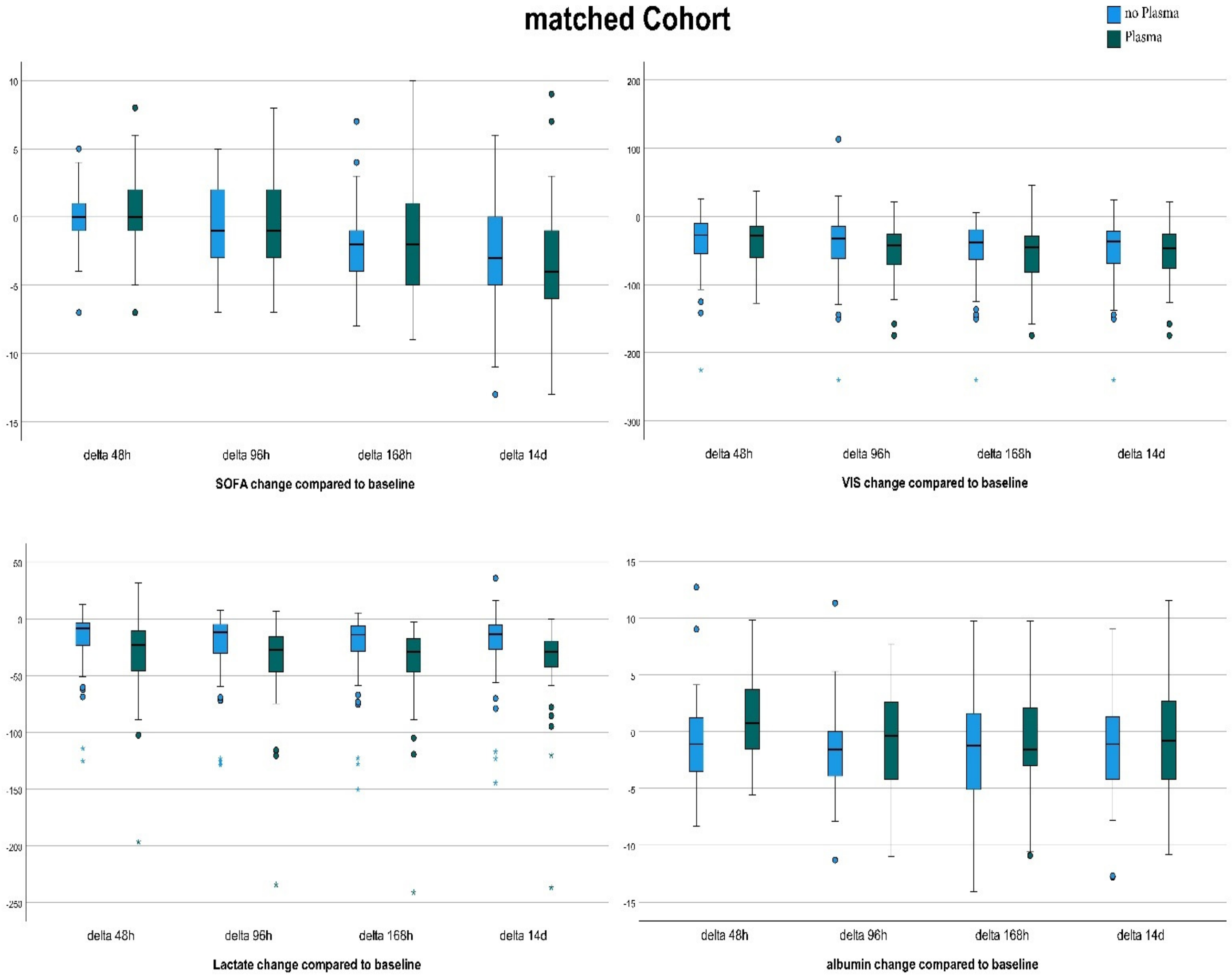

Supplement Figure S2: ***Septic shock parameters change compared to baseline in the matched cohort.*** *No statistical analysis was performed, these figures are for reference only SOFA: Sepsis related Organ Failure Assessment Score; VIS: Vasoactive Inotropic Score.*
